# Recurrent Monostotic Fibrous Dysplasia in the Mandible

**DOI:** 10.1155/2016/3920850

**Published:** 2016-05-31

**Authors:** Nilton Alves, Reinaldo José de Oliveira, Denise Takehana, Naira Figueiredo Deana

**Affiliations:** ^1^CIMA Research Group, Faculty of Dentistry, La Frontera University, 1145 Francisco Salazar Avenue, P.O. Box 54-D, 4780000 Temuco, Chile; ^2^Faculty of Sciences of Guarulhos (FACIG), 1844 Guarulhos Avenue, 7196 Guarulhos, SP, Brazil; ^3^Private Physical Therapist, 1171 Pasaje Frankfurt, 4780000 Temuco, Chile

## Abstract

Fibrous dysplasia (FD) is a condition in which normal bone marrow is replaced by an abnormal proliferation of new fibrous connective tissue. Female patient, white, 20 years old, attended the dental clinic reporting a slow increase in volume in the right mandible region over the last 5 years. She was examined by imaging: the panoramic X-ray revealed a lesion with the appearance of ground glass while the cone-beam computed tomography showed an extensive lesion in the region of the right hemimandible. The histopathological examination was compatible with fibrous dysplasia. Bone gammagraphy was indicated, plus an endocrinological study to eliminate polyostotic forms, which produced a negative result. Monostotic fibrous dysplasia in the right hemimandible was diagnosed. Conservative surgery was carried out and after 1 year recurrence of the tumour was observed. We may conclude that conservative surgery might not be the best choice for treatment for monostotic fibrous dysplasia in the mandible and that other options must be considered, such as radical surgery or the use of bisphosphonates. In our study, we may also conclude that it is very important to explain to the patient the possibility of recurrence of the lesion and the need for monitoring with periodic imaging studies.

## 1. Introduction

Fibrous dysplasia (FD) is a condition in which normal bone marrow is replaced by an abnormal proliferation of new fibrous connective tissue [1, 2].

FD is caused by the genetic mutation of the cell-surface receptor guanine nucleotide protein (G protein) [3]. The *α*-subunit of the stimulatory G protein (G_S_ *α*) activates the adenylyl cyclase, which in turn catalyzes the formation of cyclic adenosine monophosphate from ATP [4]. The gene for G_S_ *α* (GNAS1), when it suffers a mutation, is associated with the FD disorder spectrum. This mutation occurs in a somatic cell producing somatic mosaicism, in which the cells descending from the mutated cell produce abnormal characteristics, while the cells descending from the unaffected cells produce normal characteristics [5]. The increase in the activity of stimulatory G protein (G_S_) in osteoblast progenitor cells is thought to be the result of an increase in their proliferation and abnormal differentiation. Studies have established a link between the G_S_ *α* mutation and the increased production of interleukin-6 stromal cells, which promotes osteoclast activity [6]. Thus, the complex of the FD pathogenesis arises from an imbalance between bone formation and destruction [7].

It generally appears in the first or second decade of life; it is asymptomatic, progresses slowly, and affects women twice as frequently as men [1, 2]. It affects the maxilla more frequently than the mandible and may involve one (monostotic) or less commonly two or more bones (polyostotic) [1]. The signs and symptoms vary depending on the type and location of the FD and include facial deformity and asymmetry, visual alteration, auditory disability, nasal congestion and/or obstruction, pain, paraesthesia, or malocclusion [8]. The dental arch is usually maintained, although tooth displacement, malocclusion, and interference with dental eruption may occur occasionally [1].

The object of this paper is to present a case of recurrent monostotic fibrous dysplasia in the mandible region, which was studied by imaging, with incisional biopsy for definitive diagnosis. Bone remodelling was carried out to improve the patient's facial contour.

## 2. Case Report

**Figure 1:**
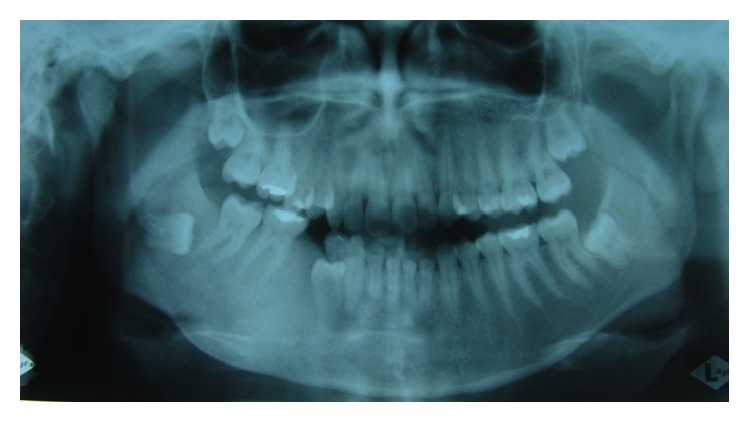
Panoramic X-ray (2005) showing alteration in the cancellous bone of the right hemimandible.

Female patient, white, 20 years old, with no systemic history, attended the dental clinic in 2005 reporting a slow, painless increase in volume in the mandible region (right side) over the last 5 years, producing facial deformity. During physical examination facial asymmetry was observed due to an increase in the size of the mandibular body on the right side, of solid consistency, pain-free, and without adenomegalies. Intraoral examination showed a hard-textured increase in the alveolar ridge on the right side; the adjacent mucous tissue was normal. A panoramic X-ray was done (Figure 1) in which alteration was observed in the pattern of the cancellous bone in the right hemimandible; it was then decided to do an incisional biopsy, resulting in a definitive diagnosis of fibrous dysplasia. Bone gammagraphy was indicated plus an endocrinological study to eliminate polyostotic forms which produced a negative result. Monostotic fibrous dysplasia in the right hemimandible was diagnosed. The treatment options were discussed jointly and it was decided to carry out monitoring with a sequence of imaging studies, with the patient being recalled for control.

**Figure 2:**
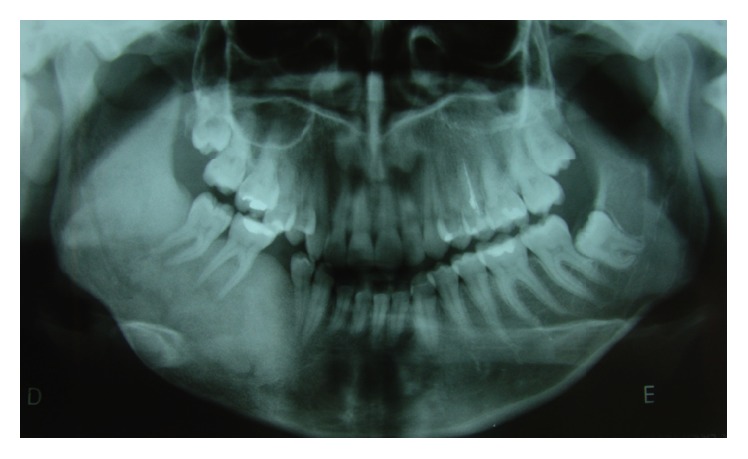
Panoramic X-ray (2009) showing radioopaque area of the image with the appearance of ground glass in the right hemimandible. It can be seen that pieces 4.5 and 4.8 have been removed.

**Figure 3:**
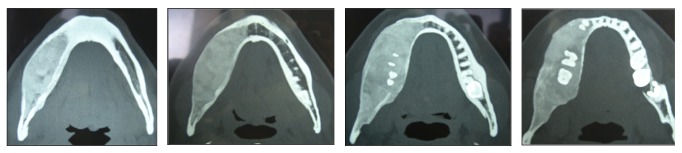
Axial sections by cone-beam computed tomography showing increase in the size of the mandibular body on the right side.

**Figure 4:**
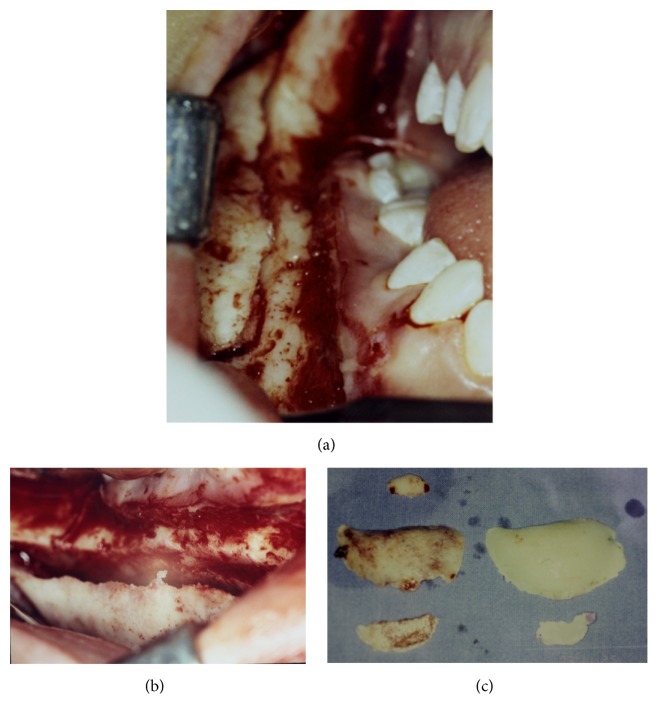
(a and b) Photographs of the surgical procedure; (c) photograph of surgical pieces removed.

**Figure 5:**
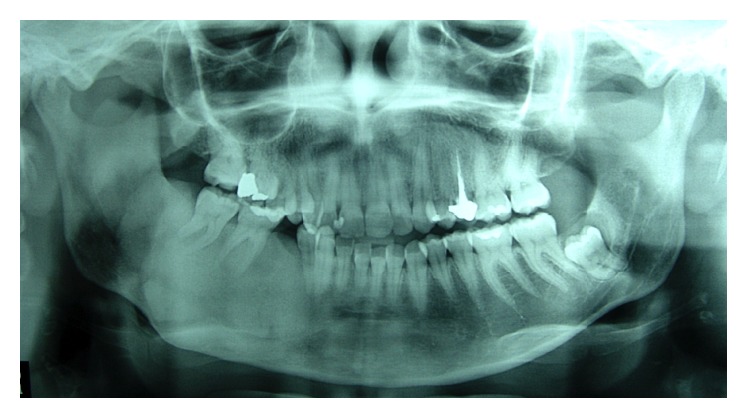
Panoramic X-ray (2013) showing radioopaque area of the image with the appearance of ground glass in the right hemimandible. Recurrence of the lesion.

**Figure 6:**
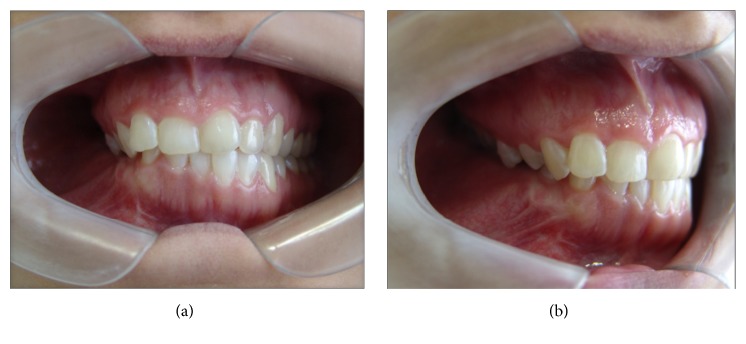
(a) Anterior and (b) anterolateral view of the intraoral region showing an increase in the alveolar ridge in the right hemimandible.

**Figure 7:**
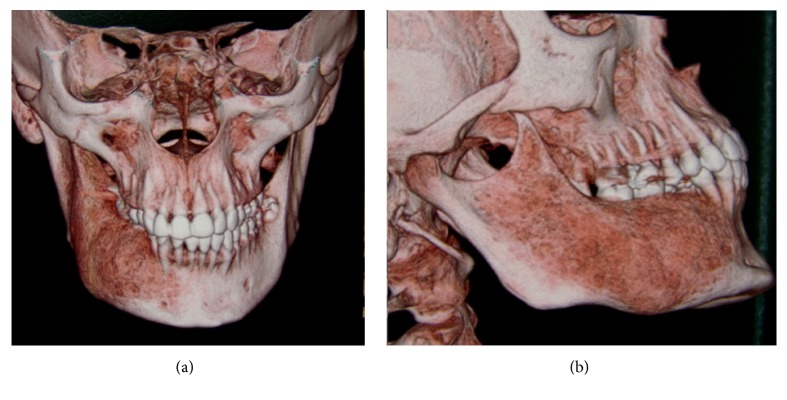
3D reconstruction by cone-beam computed tomography showing an increase in the volume of the right hemimandible. (a) Anterior view and (b) lateral view.

When the patient returned for control four years later, in 2009, she expressed interest in treating the facial deformity produced by the pathology. She reported that she had received surgical treatment to remove two teeth which were retained in the mandible on the right side. After clinical examination, a panoramic X-ray was done (Figure 2) in which it was observed that pieces 4.5 and 4.8, which were retained, had been removed. Furthermore there was a radioopaque area of the image with the appearance of ground glass in the right hemimandible. A more detailed examination by cone-beam computed tomography (CBCT) not only confirmed the existence of the bone lesion in the right hemimandible (Figure 3), but also allowed the location and extent of the tumour to be determined precisely, from which the surgical procedure indicated could be planned correctly. A conservative surgical treatment was carried out, removing some bone (Figure 4) and remodelling the region affected by the deformity. The patient was asked to return for monitoring through a sequence of imaging studies. When the patient returned for control after four years, in 2013, clinical and X-ray examination revealed recurrence of the lesion.  Figure 5 presents the panoramic X-ray showing a radioopaque area of the image with the appearance of ground glass in the right hemimandible.  Figure 6 is the intraoral appearance in which an increase of the alveolar ridge on the right side can be observed.  Figure 7 shows the 3D reconstruction by cone-beam computed tomography (anterior and lateral views) with an increase in the volume of the right hemimandible, confirming mandibular asymmetry. The patient has been advised to continue with monitoring by sequential imaging studies.

## 3. Discussion

Craniofacial bones are affected in approximately 30% of cases of FD [9]. In a retrospective study of 25 patients, the mandible was the primary site of the tumour in 76% of cases. It may appear in the premolar and molar region, that is, from the mental foramen towards the angle of the mandible; the anterior part of the mandible is least affected [2].

Asymmetry and oedema are the most common complaints when FD is found in the bones of the facial skeleton [2]. Other symptoms such as malocclusion of the teeth, pain, distortion of facial contour, alveolar abscess, and cellulitis in the face are also reported in the literature [2, 10]. In our case the patient's main complaint was facial asymmetry, but malocclusion of the teeth and distortion of facial contour were also present.

Tumours in young patients or recent tumours present a cyst-like appearance. The smallest mandibular tumour was 1.5 cm in diameter [2]. In general small tumours are unilocular while larger tumours are multilocular. The majority of lesions caused by FD presented large, irregular cancellous zones, with mottled appearance because of other masses of calcified material.

The preferred imaging examination is cone-beam computed tomography, since it offers a broader view of the extent and location of the lesion, which helps in planning the surgical procedure [11]. The most frequent X-ray characteristic of craniofacial FD is “ground glass” appearance with a fine cortex and no defined borders [12]; this was the kind of image found in examination of our patient. Ogunsalu et al. [12] propose the following categories of X-ray appearance: (1) orange peel/ground glass appearance; (2) opacification of the antrum; (3) radiolucent lesion of the mandible; and (4) varying degrees of opacification (not ground glass and not orange peel).

Hart et al. [13], in a study of 266 bone gammagraphy images in 66 patients, showed that 90% of FD lesions, regardless of site, were present before the age of 15. However the literature reports cases of adults developing FD. Ogunsalu et al. [12] studied 15 cases of patients with FD in the maxilla and mandible, reporting that in 10 cases the lesions presented initially after the age of 20 and in 2 cases at the age of 47. Pinsolle et al. [14] studied 25 patients with FD of the craniofacial bone aged between 8 and 56 years. 70–80% of cases of monostotic FD occur in the second and third decade of life [15, 16]. In our study the patient presented the initial lesion before the age of 15.

Some authors report that growth of this lesion tends to stop around puberty [17]; however other studies indicate that its progression is continuous into adulthood [18]. In our study the patient reported that growth started at puberty, contradicting the literature. Another important fact is that approximately 20–25% of these tumours continue to grow after treatment, regardless of the type of therapy applied, except if they are extirpated radically, it is impossible to prognosticate whether the FD will recur or not [2, 19].

Primary treatment of mandibular lesions always includes correction of asymmetries and deformities and/or prevention of functional problems by conservative surgery which can be carried out regardless of the size of the lesion; this is the technique indicated by many authors [2, 9, 20]. In our case we performed excision of the tumour using a conservative procedure. After 1 year growth of the lesion was observed in the same region, from which we concluded that conservative treatment was insufficient in this case. In a retrospective study of 68 patients, including 19 cases of monostotic FD and 2 cases of polyostotic FD involving the mandible, Valentini et al. [21] reported that there was no case of recurrence when radical resection of the lesion was applied; further surgery due to recurrence of the tumour was necessary in only one case of FD in a mandible which had received conservative treatment. These authors consider that radical surgery prevents recurrence and is the only option for eliminating the disease. Treatment options apart from surgery and drugs for pain control are limited, but one alternative for the treatment of FD is the use of bisphosphonates. Bisphosphonates are drugs that reduce osteoclast activity when they bind to the bone surfaces, particularly those subjected to active resorption, which acts as a biochemical barrier for bone resorption [22]. Its use in adult patients has presented good results in the control of FD, inhibiting progression of the disease once treatment has begun [23]. The use of this drug promotes reduced bone pain, a decrease in biochemical markers of bone turnover, and a radiographically apparent “refilling of osteolytic sites” in some patients [24]. However, prolonged use must be monitored because it can cause an increase in bone mineral density [23]. One important side effect caused by the use of bisphosphonates is osteonecrosis of the jaw [25, 26]. In addition, it is known that the medication remains in the skeleton for a long time and caution is recommended for use in women of child-bearing age [27]. In our study the patient presented a good health condition, but as a young female patient the decision was made not to treat with bisphosphonates.

Considering that there was recurrence in our case and that similar cases are reported in the literature, we may conclude that conservative surgery may not be the best choice for treatment for monostotic fibrous dysplasia in the mandible and that other options must be considered, such as radical surgery or the use of bisphosphonates. It is important for the dentist to analyze each case of FD, taking aspects like gender, age, recurrence, extent of the lesion, and the involvement of adjacent structures into consideration in order to choose the best treatment for each patient. In our study we may also conclude that it is very important to explain to the patient the possibility of recurrence of the lesion and the need for monitoring with periodic imaging studies.
